# Kidney biopsy adequacy and complications in children — does technique matter?

**DOI:** 10.1007/s00431-022-04464-1

**Published:** 2022-04-12

**Authors:** Chen Pettit, Roshana Kanagaratnam, Finbarr Coughlan, Nicole Graf, Deirdre Hahn, Anne Durkan

**Affiliations:** 1 University of Sydney, Sydney, NSW Australia; 2 Royal Prince Alfred Hospital, Sydney, NSW Australia; 3 University of New South Wales, Sydney, NSW Australia; 4 Department of Histopathology, The Children’s Hospital at Westmead, Sydney, Australia; 5 University of Western Sydney, Parramatta, NSW Australia; 6 Department of Nephrology, The Children’s Hospital at Westmead, Sydney, Australia

**Keywords:** Kidney biopsy, Histological diagnosis, Adequacy, Complications, Technique

## Abstract

Kidney biopsy is part of the diagnostic workup of many children with renal disease. Traditionally, a perpendicular approach to the biopsy has been used, but more recently, some proceduralists have favoured a tangential approach. It is not clear if one technique is superior with regards to tissue adequacy or complication rates. In our centre, interventional radiologists (IR) use general anaesthetic and a tangential approach, whereas paediatric nephrologists (PN) use sedation and a perpendicular approach. We examined consecutive native kidney biopsies performed between January 2008 and December 2017 for adequacy (sufficient tissue for light and electron microscopy and immunofluorescence) and examined the electronic medical records for data regarding technique and complications. IR performed 72 (29%) of the 245 native kidney biopsies, obtaining more total glomeruli (median 39 *vs* 16, *p* < 0.001) and more glomeruli per tissue core (median 13 *vs* 8, *p* < 0.001) than PN. No differences in specimen adequacy were observed between the two groups (79% IR *vs* 81% PN, *p* = 0.75) and a diagnosis could be made in 99% and 94% respectively (*p* = 0.1). A statistically lower rate of peri-nephric haematoma (28% *vs* 42%, *p* = 0.04) was detected in the IR group, but there were no significant differences in other complications. One patient required a blood transfusion (PN) and another required surgical intervention for a perinephric haematoma (IR).

*Conclusion*: IR obtained larger samples and number of glomeruli, but the overall adequacy for native kidney biopsies was good using both perpendicular and tangential techniques, with low rates of significant complications.

**Table:** 

**What is Known:** *• Kidney biopsy is integral to the diagnostic work-up of many children with kidney disease.* *• Kidney biopsy is a safe procedure with well-established complications in a minority of children.*	
**What is New:** *• Interventional radiologists had higher biopsy yield than paediatric nephrologists, possibly due to the tangential approach.* *• Biopsy adequacy rates are high using both techniques and provided a diagnosis in over 95% of cases.*

**Supplementary information:**

The online version contains supplementary material available at 10.1007/s00431-022-04464-1.

## Introduction

Percutaneous kidney biopsy (PKB) is integral to the current standard of care in the diagnosis of many renal diseases in children [1]. Different techniques for obtaining kidney tissue have evolved, with no universally accepted best method. Traditionally, a perpendicular approach has been taken, but more recently, a tangential approach has been favoured by some. The tangential approach involves the biopsy needle entering between 45 and 60° to the renal capsule, theoretically obtaining more cortical tissue, containing glomeruli, as the needle traverses a proportionally greater area of cortex. A higher rate of sample adequacy has been shown in adult settings using this technique in both native and transplant kidney biopsies [2–5]; however, it is not known if this is the case in children. The limited evidence suggests that both techniques are safe.

Increasingly, interventional radiologists are performing kidney biopsies on children; however, direct comparison between radiologist and nephrologist obtained biopsies is lacking in the paediatric setting. Several studies have compared biopsy adequacy and complication rates between nephrologists and radiologists in adult populations with discrepant results, with some studies finding no difference between the operators, whereas others found that nephrologists obtained smaller samples with fewer glomeruli [6–9].

A major problem in assessing native kidney biopsy adequacy is the heterogeneity of definition used in the published literature. To be diagnostic biopsy cores should sample adequate compartments of interest including glomeruli, arteries/arterioles, tubules, and interstitium. Samples should be examined under all available modalities — light microscopy, immunofluorescence, and electron microscopy. There is no uniformly accepted minimum number for glomeruli or other components of the native kidney biopsy sample [10]. For diseases such as membranous glomerulonephritis, a single glomerulus may be sufficient for diagnosis, but for other diseases, such as focal segmental glomerulosclerosis (FSGS), many glomeruli may be required to detect the diagnostic histopathological lesion [11, 12].

Kidney biopsies are routinely performed with ultrasound guidance. However, given the large blood flow to the organ, it is impossible to avoid sampling of small arteries or arterioles. Indeed, it is considered a useful adjunct to have histopathological imaging of the vasculature. It is therefore not unexpected that bleeding is fairly common after a biopsy. Bleeding complications mainly include haematuria and perinephric haematoma, both of which generally resolve spontaneously [13–16]. Rarely bleeding may require blood transfusion or even more rarely intervention such as angiography, embolisation, or nephrectomy [17]. Arteriovenous (AV) fistula is also reported after renal biopsy [18, 19].

The aims of this study were (i) to evaluate the overall adequacy and complication rates of native kidney biopsy in children at our institution and (ii) to compare the adequacy and safety profile between interventional radiologists (IR), who use a tangential approach, and paediatric nephrologists (PN), who use a perpendicular approach.

## Methods

This retrospective cohort study examined diagnostic renal biopsies conducted over a decade in one of the largest children’s hospital in Australia. Ethical approval was granted by the Human Research Ethics Committee (HREC) of the Sydney Children’s Hospitals Network (HREC approval: LNR/17/SCHN/474). A medical record coding search was carried out to identify all kidney biopsies performed at the Children’s Hospital at Westmead, Sydney, between 1 January 2008 and 31 Dec 2017. Only children aged 18 years or less at the time of the biopsy were included. Kidney tumour, transplant allograft, and back-table biopsies performed during transplant surgeries were excluded. Children needed at least 3-year follow-up to allow for evidence of disease progression in those with normal or non-diagnostic biopsies.

### Biopsy technique

During the 10-year study period biopsies were performed by two interventional radiologists (IR)and fifteen paediatric nephrologists (PN) including five consultant and ten trainee nephrologists. Trainee nephrologists performed biopsies independently with consultant supervision using the same perpendicular technique as consultant nephrologists. IR performed biopsies in operating theatres, usually on scheduled lists, on children undergoing general anaesthesia. Generally, these children were deemed unsuitable for sedation due to their age or co-morbidities or they were undergoing general anaesthesia for another procedure simultaneously. PN performed biopsies on lightly sedated children, using oral morphine and midazolam with inhaled nitrous oxide if required, in a procedure room in the ultrasound department. Only children with a normal coagulation profile and platelet count > 80,000 × 10^9^/L were eligible for biopsy. Desmopressin was administered to those with urea > 35 mmol/L to reduce the risk of uraemic coagulopathy. Real-time ultrasound guidance was used for all biopsies, with IR performing their own ultrasound and PN being assisted by an ultrasonographer. An 18-gauge biopsy needle was used in all cases. PN used a Bard Max Core biopsy gun (Bard Biopsy, Tempe, Arizona, USA) with a fixed penetrating depth of 22 mm. One interventional radiologist used a semi-automated Bard Mission biopsy gun in a coaxial system with an adjustable throw of 10 mm or 20 mm. The other used a Biopince Full Core biopsy gun (Argon Medical Devices, Athens, Texas, USA) with adjustable throws of 13 mm, 23 mm, and 33 mm. PN used a perpendicular entry to the kidney, whereas IR used a tangential approach of approximately 45°. At the end of the procedure, an ultrasound scan was performed. All cores obtained were sent to be examined by a histopathologist using a dissecting microscope. If the sample was thought to be inadequate, a further core(s) was requested by the histopathologist. The post-procedure urine of all children was examined to assess for haematuria for at least 6 h. Children were discharged after 6 h of bedrest if they were haemodynamically stable and had no persistent macroscopic haematuria, if there was no other indication for hospital admission. There were no major changes to the procedures used over the 10-year period.

### Data collection

Demographic and clinical data were manually retrieved from the electronic medical record for each patient, along with the designation of the biopsy operator, details of the procedure, and follow-up. All available post-procedure ultrasound reports were examined to assess for the presence of arterio-venous fistulae, but a late post-procedure ultrasound was not part of routine follow up.

Biopsy yield (the number of cores, glomeruli, and arteries obtained) were identified from the histopathology report. Biopsies were examined by at least one of three histopathologists, with consensus opinions given in many cases. In the event of missing data on the report, the slides were reviewed (by NG or FC).

### Definitions

Adequate native biopsy — sample large enough to be successfully processed for light microscopy (LM), immunofluorescence (IF), and electron microscopy (EM), with at least one whole glomerulus examined in each modality.

Obesity — defined using International Obesity Task Force Criteria, which provides BMI cut points by age and sex for obesity for children age 2 to 18 [20].

Hypertension — previously established diagnosis of hypertension as recorded in patients’ eMR.

Perinephric haematoma — any perinephric fluid collection seen on ultrasound within 48 h of biopsy.

De novo macroscopic haematuria — any new onset of macroscopic haematuria within 48 h of biopsy.

### Statistical analysis

Results are expressed as percentages, median values, and interquartile ranges. Differences of categorical data were tested using Pearson’s chi-squared test or Fisher’s exact test. Differences of continuous variables were detected using the Mann–Whitney *U* test after the normality test. Listwise deletion method was used to eliminate missing data.

The associations between potential predictors (age, sex, operator type and BMI) and primary outcomes (adequacy and glomeruli per core) were examined. When examining the association with binary outcome of adequacy, for categorical predictor groups, Pearson’s chi-squared test was used, and for continuous predictor groups, binary logistic regression was used. When examining the association with glomeruli per core, Poisson regression was used with number of glomeruli being outcome variable and number of cores being exposure variable for both categorical and continuous predictors.

All analyses were performed on IBM SPSS Statistics 24 (IBM Corp, released 2016, IBM SPSS Statistics for Windows, Version 24.0. Armonk, NY: IBM Corp). A *p* value < 0.05 was considered to be statistically significant, and all *p* values are two-tailed.

## Results

A total of 482 consecutive biopsies were performed during the study period; 237 renal mass, transplant, and back-table biopsies were excluded. Of the 245 biopsies included, 72 (29%) were done by IR, and 173 were done by PN. Subject characteristics are presented in Table 1.

**Table 1:** Subject characteristics at the time of biopsy, comparing interventional radiology and paediatric nephrology performed biopsies.

	**Radiology**	**Nephrology**	**P value**
	**n = 72**	**n = 173**	
**Age (years)**	4.4 (2.6-6.9)	12.1 (8.6-14.7)	<0.001
**Male (%)**	54%	54%	0.98
**Height (cm)**	103 (91-122)	151 (130-162)	<0.001
**Weight (kg)**	18 (15-24)	44 (29-58)	<0.001
**Obesity (%)**	17%	11%	0.24
**Hypertension (%)**	65%	54%	0.10
**Haemoglobin (g/L)**	122 (104-132)	120 (104-134)	0.67
**Platelet count (× 10**^**9**^**/L)**	353 (235-458)	275 (209-364)	<0.01
**eGFR (ml/min/1.73 m**^**2**^**)**	110 (63-161)	124 (73-155)	0.88
Indication for biopsy*			
**Non-nephrotic proteinuria**	39%	53%	<0.05
**Raised creatinine or AKI**	31%	30%	0.94
**Nephrotic range proteinuria**	32%	15%	<0.05
**Haematuria**	25%	31%	0.33
**Other**	0.0%	0.6%	0.52

*eGFR* estimated glomerular filtration rate calculated using Schwartz formula, *AKI* acute kidney injury

Results are expressed as median value (interquartile range) and percentages

Obesity defined using the International Obesity TaskForce (IOTF) reference values

*Some children had more than one indication

IR performed biopsies on younger (median age 4.4 *vs* 12.1 years, *p* < 0.001) and smaller (median weight 18.3 vs 43.9 kg, *p* < 0.001) children than PN; however, the obesity rate (17% *vs* 11%, *p* = 0.237) was not significantly different. The eGFR (using the modified Schwartz formula) and hypertension rate were not statistically different between the two groups. The most common indications for kidney biopsy were non-nephrotic range proteinuria (49%), impaired renal function (30%), nephrotic range proteinuria (20%), and macroscopic haematuria (usually in association with either proteinuria or impaired renal function) (29%). Some children had more than one indication. IR performed more biopsies on children with nephrotic syndrome (32% *vs* 15%, *p* < 0.05) as these were usually non-urgent and could be performed on a routine operating list.

### Biopsy yield

IR obtained a median of 3 (interquartile range 2–4) kidney cores compared to 2 (interquartile range 2–3) by PN. They also had more glomeruli per core (median 13 *vs* 8, *p* < 0.001). Overall IR obtained more total glomeruli (median 39 *vs* 16, *p* < 0.001), as shown in Table 2.

**Table 2:** Biopsy yield – median value (interquartile range) of passes, cores, glomeruli per core, glomeruli obtained by interventional radiologists compared with paediatric nephrologists.

	**Radiology**	**Nephrology**	**P value**
	**n=72**	**n=173**	
**Passes**	3 (2-4)	2 (2-3)	<0.001
**Cores**	3 (2-4)	2 (2-3)	<0.001
**Glomeruli per core**	13 (8-19)	8 (5-10)	<0.001
**Glomeruli **	39 (25-57)	16 (10-22)	<0.001

### Biopsy adequacy

The overall adequacy rate of native biopsy was 80%, with no significant difference between IR and PN (79% IR *vs* 81% PN, *p* = 0.75). Using an alternative definition of “being able to make a diagnosis,” the overall adequacy increased to 95%, again with no significant difference between IR and PN (99% *vs* 94%, *p* = 0.10). Comparing trainee (*n* = 107) and consultant nephrologist biopsies (*n* = 48), there was no significant difference in adequacy, yield, or complication rate. In 18 cases, it was unclear what grade of physician had performed the biopsy.

### Predictors of biopsy yield and adequacy

There was no evidence of association between any of the predictors with adequacy in univariable model therefore multivariable model was not used (Supplementary table 1).

There was strong evidence of association between glomeruli per core and each of the predictors in univariable models (Supplementary table 2). In the multivariable model, IR and younger age maintained their association with larger number of glomeruli per core (*p* < 0.001) but sex and BMI *z*-score were not associated with number of glomeruli per core (Supplementary table 3).

### Post-biopsy diagnosis

The most common post-biopsy renal diagnoses were minimal change disease (19%), lupus nephritis (18%), and IgA vasculitis/IgA nephropathy (17%), as shown in Supplementary Table 4.

A diagnosis is particularly important in those children who develop progressive renal impairment and the potential need of a kidney transplant, as families need to be counselled about the risk of disease recurrence. We therefore analysed more thoroughly the data of the children with non-diagnostic abnormalities. The results of these 12 children are shown in Table 3, along with the data of those who had normal biopsies. Two-thirds of those with non-diagnostic abnormalities presented with advanced renal impairment, and accordingly, five had non-reversible histopathological changes of end stage renal failure. Of these five, one was subsequently found to be ANCA positive and one had a genetic form of FSGS. One child underwent a second biopsy and was diagnosed with Alport’s syndrome, having not had sufficient tissue for EM on the first biopsy. The primary diagnosis in six who progressed to renal replacement therapy remained elusive. In contrast, none of the 10 children with no histological abnormalities had evidence of disease progression, suggesting that important anomalies were not missed due to either sampling or human error.

**Table 3:** A comparison of normal and non-diagnostic biopsy characteristics.

	**Normal biopsy**	**Non-diagnostic biopsy**
	**n=10**	**n=12**
**Operator**		
PN	8 (80%)	11 (92%)
**Indication**		
Proteinuria	6 (60%)	1 (8.3%)
Haematuria	3 (30%)	1 (8.3%)
eGFR <40ml/min/1.73m^2^ Other	0 (0%) 1 (10%)	8 (67%)* 2 (17%)
**Adequacy**		
Light microscopy	10 (100%)	12 (100%)
Immunofluorescence	10 (100%)	8 (67%)
Electron microscopy	9 (90%)	10 (83%)
**Median glomeruli (range)**	18 (12-70)	6 (2-27)
**Disease progression**	0 (0%)	9 (75%)
**Repeat biopsy required **	0 (0%)	1 (8.3%)^a^

Normal biopsy – no identified histopathological abnormalities

Non-diagnostic biopsy – non-diagnostic histopathological abnormalities reported in the biopsy

*5 had “end stage kidney disease” on biopsy, 1 was subsequently found to be ANCA positive and another had a homozygous mutation in *PLCE-1*

^a^Alport’s diagnosed on electron microscopy

### Post-procedural complications

Biopsies performed by IR resulted in significantly fewer perinephric haematomas (28% IR and 42% PN, *p* = 0.04). There were no significant differences in other complication rates. See Table 4.

**Table 4:** A comparison of complications arising following biopsies performed by interventional radiologists and paediatric nephrologists.

	**Radiology**	**Nephrology**	**P value**
	**n = 72**	**n = 173**	
**Perinephric haematoma**	20 (28%)	72 (42%)	0.04
**De novo macroscopic haematuria**	12 (17%)	30 (17%)	0.90
**Unplanned overnight admission**	13 (18%)	26 (15%)	0.56
**Transfusion**	0 (0%)	1 (0.6%)	1.00
**Biopsy-related infection**	2 (2.8%)	0 (0%)	0.09
**Arteriovenous fistula**	0 (0%)	0 (0%)	-

Numbers of individual complications are given with percentages in brackets

One case in the IR group required an emergency angiogram with wire manipulation and platelets transfusion because of a large perinephric haematoma. This patient returned approximately 1 week later with a urinary tract infection. Another case in the IR group was found to have an intrarenal haemorrhage 4 days post biopsy and developed fever with rigors 2 days later, but urine culture was negative. The haemorrhage resolved spontaneously. Blood transfusion was required in one case in the PN group in association with de novo macroscopic haematuria. No AV fistulae were detected during the period of study.

## Discussion

This study shows that in 80% of cases of native kidney biopsy, an adequate sample was obtained based on the availability of tissue for LM, IF, and EM. In 95% of biopsies, a diagnosis was possible. Obtaining adequate tissue is crucial to maximising the diagnostic accuracy and usefulness of the procedure. Unlike in transplant biopsies where definition of adequacy has been well established since the 1997 Banff criteria [12], a major issue for native biopsies is the lack of a universally accepted definition of adequacy and this has resulted in different definitions being used in the published literature, making comparison between studies difficult. Our primary definition, of sufficient tissue to be examined by all three modalities, may not be adequate for some conditions such as FSGS, where a large sample size ideally from the cortico-medullary region is preferred. Some studies have extended the Banff criteria to both native and transplant biopsies and have demonstrated adequacy rates of 93–98% [21, 22]. Using this same definition, 84% of our biopsies were adequate. One major difference between our study and the previous studies is the number of operators. Our hospital is a tertiary training centre and the majority of our biopsies were done by trainee nephrologists, perhaps resulting in more variability than in a centre where one person performs all the procedures, though it should be noted that we did not find a difference in adequacy rates based on the grade of the operator.

The largest paediatric dataset is from a survey across 11 UK paediatric nephrology centres, demonstrating a combined adequacy rate of 97% in native and transplant cases using adequacy criteria defined as “ ≥ 10 glomeruli OR histopathologist able to make a clear diagnosis on limited number of glomeruli using all modalities” [14]. This broad definition would include many that we considered to be inadequate and makes a direct comparison difficult.

When comparing the IR and PN biopsies, there were inevitable differences in the baseline characteristics of the two groups, as IR tended to perform biopsies on younger children who required general anaesthesia or those for whom sedation was inappropriate. The rates of obesity and formally diagnosed hypertension were similar between two groups. The differences in platelet count can be largely explained by physiology. Interventional radiologists obtained larger kidney samples, as observed in more glomeruli per core of tissue. The tangential approach used may contribute to this, as the needle will pass through a wider area. This approach has been validated in adult studies to achieve high sampling adequacy (95–99%), with low complication rates, yet in general, nephrologists are taught to do a perpendicular approach, as they are taught by colleagues who only know this method [2–5]. Another potential contributing factor to IR’s larger cores could be the relative needle vs body size — IR and PN both used 18 gauged biopsy needles but IR performed biopsies on younger and smaller children, with younger age proving to be statistically significant in the multivariable regression analysis. IR also took more cores of tissue-perhaps more than needed. It could be argued that two cores in most cases would have been sufficient, or that the number of cores be tailored to the anticipated histological diagnosis such that more tissue is obtained when FSGS is a differential diagnosis. It is important to note that although all samples were examined using a dissecting microscope by a histopathologist, this involved the transportation of the specimens to the histopathology laboratory, and therefore, the operator would visually assess the samples prior to transportation to gauge if a further core was necessary. We did not have documentation of whether additional cores were obtained at the request of the histopathologist or at the discretion of the operator.

There were a few other confounding factors more in favour of IR. General anaesthetics takes patient movement out of the equation, leaving only kidney movement during respiration. In experienced hands, real-time tracking of the needle trajectory could be easier with self-guided ultrasound than sonographer-directed ultrasound. It is also worth noting IR tended to perform elective biopsies on scheduled lists and PN tended to perform more urgent biopsies. Like other elective surgeries, elective biopsies tend to have less complications [23].

The finding of a normal biopsy can be concerning as it may imply that insufficient tissue was obtained to see any abnormalities. Reassuringly, we found that in the majority of these cases, the indication for biopsy was mild proteinuria and over the minimum 3-year follow-up period, none of those with normal biopsies had disease progression. On the other hand, those with non-diagnostic abnormalities often presented late, with evidence of severe renal impairment. These biopsies had fewer glomeruli, and this may be due to loss of identifiable glomeruli secondary to marked sclerosis and atrophy. The majority of these non-diagnostic biopsies were performed by PN as they were usually done as emergency procedures, frequently out of normal working hours.

The published rates of de novo post-biopsy macroscopic haematuria vary between 1.5 and 20% and transfusion requirement between 0.1 and 0.9% [13–15]. Our overall rate of de novo haematuria (17%) and transfusion rate (0.4%) are within the ranges suggested by these studies. Post-biopsy bleeding has been associated with uraemia, coagulation disorders, low platelet level, and high blood pressure [21, 24]. Although bleeding is well-recognised in association with uraemia [25, 26], the pathophysiology is not completely understood, but is likely to involve platelet dysfunction and abnormal platelet-endothelial interactions [27, 28].

Ultrasonographers documented the presence of perinephric haematomas for nephrologists, whereas radiologists made their own documentations and there was perhaps a bias to not reporting smaller haematomas that were deemed insignificant. The exact dimensional measurements were often omitted, but we postulate that large symptomatic haematomas were rare given that only one case required angiographic intervention. Our rate of perinephric haematoma was comparable to that of a Norwegian paediatric cohort [13]. One previous paediatric study identified male gender and low weight-for-height as risk factors for perinephric haematoma; however, we did not find similar associations [15].

No post-procedural AV fistulae were detected throughout the period studied. Other studies reported rates of 0.1–8.3%, with a large number resolving spontaneously [18, 25]. Given that surveillance for AV fistula is not routine in our practice, it is possible that an asymptomatic AV fistula may have been undetected.

Kidney biopsy is generally a day procedure at our institution, and this practice is supported by the low numbers of unplanned overnight hospital admissions. Other groups have similarly found kidney biopsy to be a safe day-case procedure, with improved cost effectiveness and enhanced patient satisfaction [29].

There are a number of limitations to this single centre study, and the results may not be generalisable to all paediatric centres. One major limitation is the differences in the baseline characteristics, mainly age, of the two groups. IR had larger biopsy yield than nephrologists, possibly due to the radiologists’ tangential approach, though patient immobility with general anaesthesia and self-directed real time ultrasound may also have contributed. We cannot therefore separate the effects of the tangential approach, operator guided ultrasound, and general anaesthetic in contributing to larger IR samples. Another limitation is given the number of paediatric nephrology proceduralists (fifteen); it was unclear whether there were any significant outliers given that individual operator adequacy and complication rates were not assessed. Lastly, as a late post-procedure ultrasound is not routine in our centre, we may have missed small AVFs that were not clinically significant.

## Conclusion

This retrospective cohort study found that both PN and IR obtained adequate kidney biopsy samples in the large majority of cases. IR had larger biopsy yield than PN, and their tangential approach is likely to be a factor; however, the effects of general anaesthesia and self-guided ultrasound could also be contributory. For diagnostic purposes, we cannot recommend one technique over the other, given the similar rates of diagnosis and safety profiles.

## Supplementary information

Below is the link to the electronic supplementary material.

Supplementary file1 (DOCX 24 KB)

## Data Availability

The raw data and codes are freely available to anyone requesting it from the corresponding author.

## Abbreviations

AKI: Acute kidney injury
AV: Arterio-venous
BMI: Body mass index
eGFR: Estimated glomerular filtration rate
EM: Electron microscopy
FSGS: Focal segmental glomerulosclerosis
GN: Glomerulonephritis
HREC: Human research ethics committee
IF: Immunofluorescence
IGAV: Immunoglobulin A vasculitis
IR: Interventional radiologist
LM: Light microscopy
PKB: Percutaneous kidney biopsy
PN: Paediatric nephrologist
