# Supplementary material for: Kidney biopsy adequacy and complications in children — does technique matter?
Source: Eur J Pediatr. 2022 Apr 12;181(7):2677–84. doi: 10.1007/s00431-022-04464-1 (PMC9192435; doi:10.1007/s00431-022-04464-1)
Supplement: Supplementary file 1 — Supplementary file1 (DOCX 24 KB) [file 431_2022_4464_MOESM1_ESM.docx]

| Variable | OR (95% CI) |
| --- | --- |
| Age  (per year increase) | 0.99 (0.93-1.06) |
| BMI z score  (per unit increase) | 0.88 (0.67-1.14) |
| Sex |  |
| Male | ref |
| Female | 0.90 (0.48-1.69) |
| Operator |  |
| IR | ref |
| Nephrologist | 1.12 (0.56-2.21) |

**Supplementary Table 1.** Univariate analysis of potential predictors of biopsy adequacy

OR – odds ratio, CI – confidence limits, IR – interventional radiology

| Variable | IRR (95% CI) |
| --- | --- |
| Age  (per year increase) | 0.95 (0.94-0.95) |
| BMI z score  (per unit increase) | 1.04 (1.02-1.06) |
| Sex |  |
| Male | ref |
| Female | 0.91 (0.86-0.96) |
| Operator |  |
| IR | ref |
| Nephrologist | 0.59 (0.56-0.62) |

**Supplementary Table 2**. Univariate analysis of potential predictors of glomeruli per core of kidney tissue.

OR – odds ratio, CI – confidence limits, IR – interventional radiology

| Variable | IRR (95% CI) | P-value |
| --- | --- | --- |
| Age  (per year increase) | 0.96 (0.96-0.97) | <0.01 |
| BMI z score  (per unit increase) | 0.99 (0.97-1.02) | 0.61 |
| Sex |  | 0.89 |
| Male | ref |  |
| Female | 1.00 (0.95-1.06) |  |
| Operator |  | <0.01 |
| IR | ref |  |
| Nephrologist | 0.72 (0.67-0.77) |  |

**Supplementary Table 3.** Multivariate Poisson regression of potential predictors of glomeruli per core of kidney tissue.

IRR – incidence rate ratio, CI – confidence limits, IR – interventional radiology

|  | Radiology n(%) | Nephrology n(%) |
| --- | --- | --- |
| Total | **72** | **173** |
| Minimal change disease | 19 (26.4) | 28 (16.2) |
| IgAV/IgA nephropathy | 12 (16.7) | 30 (17.3) |
| Lupus nephritis | 2 (2.8) | 42 (24.3) |
| FSGS | 9 (12.5) | 9 (5.2) |
| Other GN | 7 (9.7) | 24 (13.9) |
| Alport’s syndrome | 4 (5.6) | 7 (4.0) |
| Nephronophthisis | 4 (5.6) | 6 (3.5) |
| Normal | 2(2.8) | 8 (4.6) |
| Non-diagnostic | 1 (1.4) | 11 (6.4) |
| Other | 12 (16.7) | 8 (4.6) |

Supplementary table 4. Post biopsy diagnosis in biopsies obtained by interventional radiologists and paediatric nephrologists.
IgAV- Immunoglobulin A vasculitis (previously Henoch-Schönlein Purpura), FSGS – Focal segmental glomerulosclerosis, GN –glomerulonephritis
